# Synthesis of an insulin-loaded mucoadhesive nanoparticle designed for intranasal administration: focus on new diffusion media

**DOI:** 10.3389/fphar.2023.1227423

**Published:** 2023-08-28

**Authors:** Tahereh Jamshidnejad-Tosaramandani, Soheila Kashanian, Isaac Karimi, Helgi B. Schiöth

**Affiliations:** ^1^ Nanobiotechnology Department, Faculty of Innovative Science and Technology, Razi University, Kermanshah, Iran; ^2^ Laboratory for Computational Physiology, Department of Biology, Faculty of Science, Razi University, Kermanshah, Iran; ^3^ Department of Surgical Sciences, Division of Functional Pharmacology and Neuroscience, Uppsala University, Uppsala, Sweden; ^4^ Sensor and Biosensor Research Center (SBRC), Faculty of Chemistry, Razi University, Kermanshah, Iran

**Keywords:** intranasal drug delivery, mucoadhesive nanoparticles, chitosan nanoparticles, insulin, poly-electric complexation

## Abstract

Intranasal administration is a drug delivery approach to provide a non-invasive pharmacological response in the central nervous system with relatively small peripheral side effects. To improve the residence time of intranasal drug delivery systems in the nasal mucosa, mucoadhesive polymers (e.g., chitosan) can be used. Here, insulin-loaded chitosan nanoparticles were synthesized and their physiochemical properties were evaluated based on requirements of intranasal administration. The nanoparticles were spherical (a hydrodynamic diameter of 165.3 nm, polydispersity index of 0.24, and zeta potential of +21.6 mV) that granted mucoadhesion without any noticeable toxicity to the nasal tissue. We applied a new approach using the Krebs–Henseleit buffer solution along with simulated nasal fluid in a Franz’s diffusion cell to study this intranasal drug delivery system. We used the Krebs–Henseleit buffer because of its ability to supply glucose to the cells which serves as a novel *ex vivo* diffusion medium to maintain the viability of the tissue during the experiment. Based on diffusion rate and histopathological endpoints, the Krebs–Henseleit buffer solution can be a substituent solution to the commonly used simulated nasal fluid for such drug delivery systems.

## 1 Introduction

Intranasal (IN) drug delivery is a promising strategy to enhance the absorption of poorly penetrating active ingredients, such as proteins and small molecules, into the brain (Inoue et al., 2020; Veronesi et al., 2020). Neurodegenerative diseases often present a significant challenge for drug development due to the presence of multiple barriers, including the blood–brain barrier (BBB) and the blood–cerebrospinal fluid barrier (Bahadur et al., 2020). However, the IN route of administration offers a direct transport of drugs to the brain, bypassing these barriers (Warnken et al., 2016; Fonseca et al., 2021; Pandey et al., 2021; Rabiee et al., 2021; Agrawal et al., 2022; Bahadur and Jha, 2022; Formica et al., 2022; Vitore et al., 2023). Although there are still limitations to be considered, intranasal drug delivery is a promising delivery method for the treatment of neurological disorders (Fonseca et al., 2021). This approach takes advantage of the nasal epithelium, which provides a direct connection between the central nervous system (CNS) and the external environment (Keller et al., 2022). Moreover, mucoadhesive polymers can prolong drug retention time in the nasal mucosa, enhancing brain drug permeability compared to conventional systemic approaches (Yu et al., 2019; Wilson et al., 2021).

The IN route of administration for mucoadhesive-based or coated drug delivery systems (DDSs) offers a non-invasive and safe strategy for drug delivery to the CNS while reducing the correlated side effects of systemic drug delivery (Agrawal et al., 2018; Baboota and Ali, 2022; Moradi and Dashti, 2022). Chitosan (Cs) is a mucoadhesive biopolymer that has garnered attention for use in IN-DDSs due to its unique physical and chemical properties (Aderibigbe and Naki, 2019; Tashima, 2020). Cs is a biodegradable, biocompatible, and cationic biopolymer that can interact with negatively charged mucosal surfaces (Aajami et al., 2019). Its hydrophilic nature allows it to form a gel-like structure upon hydration (Pita-López et al., 2021), enabling it to create a strong physical bond with the mucosal surfaces and making it a reliable candidate for IN-DDSs (Sadeghi AMM. et al., 2008; Mura et al., 2022). Once an IN-DDS is developed, several experiments are required to study the interfering factors that affect the rate and extent of drug diffusion, mimicking the normal physiology of drug access to the brain (Bartos et al., 2021; Hafner, 2022).

*Ex vivo* experiments are valuable tools to study the function and behavior of biological systems in a controlled and simplified manner before proceeding to *in vivo* and clinical studies (Shi et al., 2019). Excised tissues, in particular, provide a means of evaluating the diffusion and toxicity of intranasal drug delivery systems (IN-DDSs) (Erdő et al., 2018). However, reliable diffusion investigations require the use of healthy and live tissues (Ladel et al., 2019; Salade et al., 2019; Berkhoff, 2023), as the metabolic stability and permeation mechanisms are highly dependent on tissue cell viability (Djupesland, 2013; Kolanjiyil et al., 2019; Shim et al., 2019). Currently, despite the wide range of therapeutics being developed as IN formulations, there is no validated protocol to test the diffusion of nasal excised tissues, which raises concerns about potential tissue damage that could adversely affect the diffusion rate (Williamson et al., 2019). Therefore, there is a need for more reliable methods to simulate drug penetration through the nasal epithelium.

The nasal tissue is a thin, porous, and highly vascularized epithelium that makes it a suitable site for local, systemic, and brain drug delivery (Bajracharya et al., 2019). Currently, the receptor compartments in Franz’s cells in *ex vivo* IN-DDS studies are filled with either simulated nasal fluid (SNF) or phosphate-buffered saline solution (PBS) (Chatzitaki et al., 2020; Fahmy et al., 2020; Khunt et al., 2020; Chin et al., 2021). While these solutions mimic the extracellular local microenvironment of the nasal cavity, they cannot guarantee tissue viability during the experiment, which may alter cellular transport (Carneiro et al., 2019). Diffusion plays an important role in the exchange of many different chemicals in all living tissues. However, following tissue death, the rate of diffusion may be influenced by changes in the local pH, glucose consumption, and cellular energy availability (Kia’i and Bajaj, 2019). While conducting *ex vivo* studies, factors such as tissue preparation method, experiment duration, experiment temperature, equipment used, and diffusion solution employed, among others, must be taken into consideration (Salade et al., 2019). Therefore, it is crucial to explore and develop alternative solutions that could be used to improve tissue preparation for conducting these experiments.

In this context, the Krebs–Henseleit buffer (KHB) solution is a widely used medium for *ex vivo* studies of drug diffusion across gastrointestinal membranes (Li et al., 2023; O'Shea et al., 2022). It is used to provide nutrients to the tissue and mimic the ionic composition of tissue fluid, thereby maintaining osmolality and pH constancy of biosamples (Tambe et al., 2019; Xu et al., 2021). The KHB also helps in preserving tissue viability, which allows for accurate measurements of active transport efficiency (Minasian et al., 2013; Neyshaburinezhad et al., 2020; Britto‐Júnior et al., 2021). Apart from its use in such physiological and biochemical studies, the KHB finds its application in tissue slice experiments, organ perfusion, and BBB research (Sekhar et al., 2019; Buchko et al., 2020; Pearen et al., 2020). The use of the KHB in *ex vivo* experiments provides stability to experimental conditions, which is relevant to the physiological conditions of living cells in biological samples and is readily available in laboratory settings (Berkhoff, 2023). These factors enable consistent, accurate, and relevant results, making it an ideal medium for *ex vivo* experimentation.

This study aimed to develop and characterize a stable, safe, biocompatible, effective, and mucoadhesive insulin-loaded Cs-based DDS for IN administration. Moreover, it sought to design a more valid *ex vivo* experiment with a special focus on the KHB as a new diffusion medium that can maintain tissue viability to monitor insulin penetration efficacy more accurately. To prevent adverse effects on the nasal epithelium, an isotonic formulation with a stabilized pH value of 5.5 ± 0.5 was used, conforming to the European Pharmacopeia criteria for nasal preparations (Monograph, 2017). This is the first study to compare the use of the SNF and KHB in Franz’s cell for the diffusion of insulin-loaded Cs-NPs through the ovine nasal epithelium (OVINAE).

## 2 Materials and methods

### 2.1 Chemicals

Low-molecular-weight Cs (Mw < 100 kDa), potassium dihydrogen phosphate (KH2PO4), sodium hydroxide (NaOH), hydrochloric acid (HCl), sodium chloride (NaCl), magnesium sulfate (MgSO4), potassium chloride (KCl), sodium bicarbonate (NaHCO3), calcium chloride (CaCl2), D-glucose, potassium bromide (KBr), and acetic acid were of analytical grade, and high-performance liquid chromatography (HPLC)-grade double-deionized water (DDW), methanol, and acetonitrile were purchased from Sigma-Aldrich, St. Louis, United States. The active pharmaceutical ingredient form of human insulin was kindly provided by Ronak Daroo Co. (Tehran, Iran) as a gift.

### 2.2 Methods

#### 2.2.1 Preparation of insulin-loaded Cs-NPs

Insulin-loaded Cs-NPs were synthesized according to the poly-electric complexation (PEC) method (Sadeghi A. et al., 2008). In brief, Cs was dissolved in acetic acid (1% v/v) and stirred at room temperature. The pH of the Cs solution was adjusted to 5.5 ± 0.1 by adding NaOH (1.0 M). The insulin solution was prepared in HCl (0.01 N), and the pH was adjusted to 8.0 ± 0.1 by NaOH (1.0 M). The best concentration and the volume ratios of Cs/insulin were determined according to a set of optimization experiments, with the one-factor-at-a-time method, based on the ranges reported previously (Sarmento et al., 2006; Sadeghi A. et al., 2008; Shamsa et al., 2018; Chen et al., 2019).

The insulin-loaded Cs-NPs were separated from the aqueous medium containing non-associated insulin through centrifugation at 20000 rpm for 20 min at 4°C, and the supernatant was submitted to determine encapsulation efficiency (EE) and loading capacity (LC). The concentration of free drugs in the supernatant was measured by reversed-phase HPLC (Agilent, Germany) at the wavelength of 215 nm, mobile phase: sodium sulfate buffer solution and acetonitrile, flow rate 0.75 mL/min, column: C18, 4 µm. The EE and LC of Cs-NPs were calculated using equations (A) and (B), respectively, given as follows. All measurements were performed in triplicate and averaged:

$$EE=\frac{Total\;drug-free\;drug}{Total\;drug}\times 100,$$ (A)

$$DL=\frac{Total\;drug-free\;drug}{Nanoparticle\;weight}\times 100.$$ (B)

#### 2.2.2 Characterization of insulin-loaded Cs-NPs

The physicochemical characteristics of NPs were assessed using dynamic light scattering (DLS; Malvern, Worcestershire, UK) for particle size, polydispersity index (PDI), and zeta potential (ζ). The sample volume used for the analysis was kept constant at 1 mL. To further analyze the surface morphology and to verify the DLS information, NPs were evaluated by using a scanning electron microscope (SEM; TESCAN, MIRA III model, Czech Republic) at an operating voltage of 30 kV. For microscopy, 50 µL of 1:10 diluted solution of insulin-loaded Cs-NPs with DDW was spread on a cover slide and dried using the vacuum desiccator in less than an hour. Thereafter, NPs were sputter-coated with a gold layer and observed under a SEM. Furthermore, the air-dried Cs-NPs were separated from free insulin by centrifugation (*vide supra*) and evaluated by Fourier-transform infrared (FTIR) spectroscopy (Bruker, Germany). Free insulin, Cs polymer, and insulin-loaded Cs-NPs were taken with KBr pellets on FTIR, and their spectra were analyzed to structure characterization.

In this continuum, *in vitro* cumulative release profiles for insulin in SNF (pH 6.5 ± 0.1), from 0 to 240 min, at 33°C ± 2°C (nasal cavity temperature), were diagrammed based on the *sample and separate method* using a centrifuge (Sigma, United States) (Weng et al., 2020). In the predetermined time intervals (15, 30, 60, 120, and 240 min), 200 µL of the supernatant following the centrifugation at 18000 rpm for 20 min at 4°C was collected. These samples were assayed via HPLC (Agilent, Germany) in the aforementioned conditions to determine the concentration of insulin at each time interval. Withdrawn samples were replaced with the same amount of fresh SNF each time to keep the experiment volume constant. To study the kinetics of drug release from Cs-NPs, the data were plotted in various kinetic models, i.e., the zero-order, first-order, Korsmeyer–Peppas, and Higuchi’s model (Paarakh et al., 2018).

#### 2.2.3 *Ex vivo* diffusion evaluation of insulin-loaded Cs-NPs

The diffusion of both insulin solution and insulin-loaded Cs-NPs across the OVINAE was evaluated using Franz’s diffusion cell while the mucosal surface faced the donor compartment (Sood et al., 2014). The Cs-NPs, equivalent to 4 mg of insulin, were poured on the membrane into the donor compartment previously incubated with the KHB for 10 min. Fresh diffusion media (SNF and KHB, pH 6.5 and pH 7.4, respectively) were used in the receptor compartments and maintained at 33°C ± 2°C under constant stirring. The medium (200 μL) was withdrawn serially from a receptor compartment in predetermined time intervals (15, 30, 60, 120, and 240 min). Withdrawn samples were replaced with the same quantity of fresh relevant buffer solution and assayed using HPLC (Agilent, Germany) at 215 nm. The cumulative diffusion was plotted and analyzed using mathematical kinetics models.

#### 2.2.4 Evaluation of SNF and KHB toxicity on the OVINAE

The OVINAE was obtained from a local butchery (Kermanshah, Iran) within 30 min of sacrificing the animal. Once decapitated at a local butchery, the OVINAE was soaked in the KHB (pH 7.4). The mucotoxicity studies on the OVINAE were conducted to evaluate the effects of the KHB on the tissue structure in comparison to those of the SNF (Seju et al., 2011). After the diffusion experiment with both buffer solutions, the tissues were carefully removed from Franz’s cells fixed in formalin solution (10% V/V) for 24 h, stained with hematoxylin–eosin (HE), and implanted in paraffin blocks for section preparation. Histological sections (5–6 μm) were observed under a light microscope (Olympus, Japan). To determine the effect of buffer solutions on the nasal tissue, the toxicity indicators such as the epithelial detachment (ED), mural destructive lesions of venules and arteries, and the intercellular space widening (ISW) of the tissue were considered as endpoints.

#### 2.2.5 Evaluation of the toxicity of insulin-loaded Cs-NPs on the OVINAE

Last but not the least, mucotoxicity studies on the OVINAE were performed to ensure the biocompatibility of the synthesized Cs-NPs (Seju et al., 2011). We used PBS (pH 6.8 ± 0.1) as a negative control (NC), isopropyl alcohol as a positive control (PC), and insulin-loaded Cs-NP solution (pH 6.00 ± 0.1) as a test sample (Ramreddy and Janapareddi, 2019). The ∼1 cm^2^ OVINAE was incubated with the test samples and negative and positive controls for 1 h. Then, histological sections were prepared and evaluated under the light microscope (Olympus, Japan) as previously mentioned.

#### 2.2.6 Statistical analysis

Data were analyzed using SPSS version 20 (SPSS Inc., Chicago, III., United States) and shown as means ± SEM (standard error of the mean). An independent-sample *t*-test was performed to show the differences between tissues mounted on Franz’s diffusion cells filled with the KHB and SNF. The one-way analysis of variance (ANOVA) was employed to show mucotoxicity in NPs, control NC, and PC groups, and *post hoc* Tukey’s HSD was pursued when ANOVA indicated a significant difference (*p* < 0.05). In all statistical evaluations, a value of *p* < 0.05 was considered as the significant level.

## 3 Results

### 3.1 Preparation of insulin-loaded Cs-NPs

Insulin-loaded Cs-NPs were successfully prepared according to the PEC method (Sadeghi A. et al., 2008). The insulin-loaded Cs-NPs were obtained upon the addition of different volumes of the insulin solution to the Cs solution in a dropwise manner. The concentration of both Cs and insulin solutions was adjusted at 1 mg/mL, and the volume ratio of Cs/insulin was optimized at 1:0.75, as the higher insulin concentration resulted in the aggregation of the particles. The insulin’s EE and DL of Cs-NPs were found to be 69.05% and 27.97%, respectively. The final pH of the insulin-loaded Cs-NP solution was recorded at 6.00 ± 0.1.

### 3.2 Characterization of insulin-loaded Cs-NPs

**FIGURE 1:**
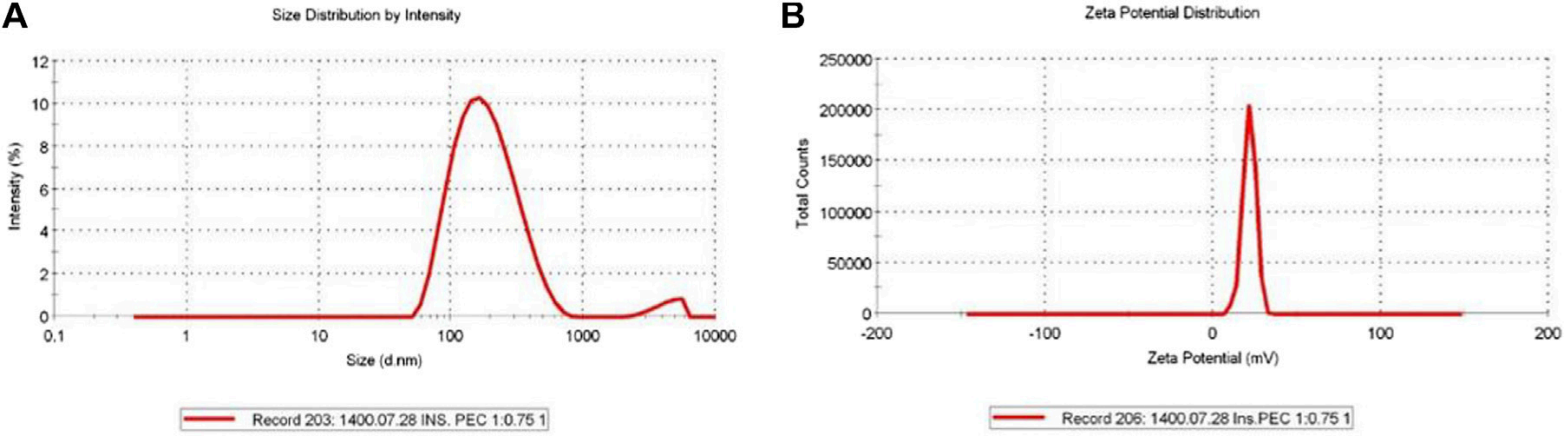
**(A)** Insulin-loaded chitosan nanoparticles’ hydrodynamic diameter (165.3 nm), polydispersity index (0.24), and **(B)** zeta potential (+21.6 mV) in a suitable range for intranasal administration.

**FIGURE 2:**
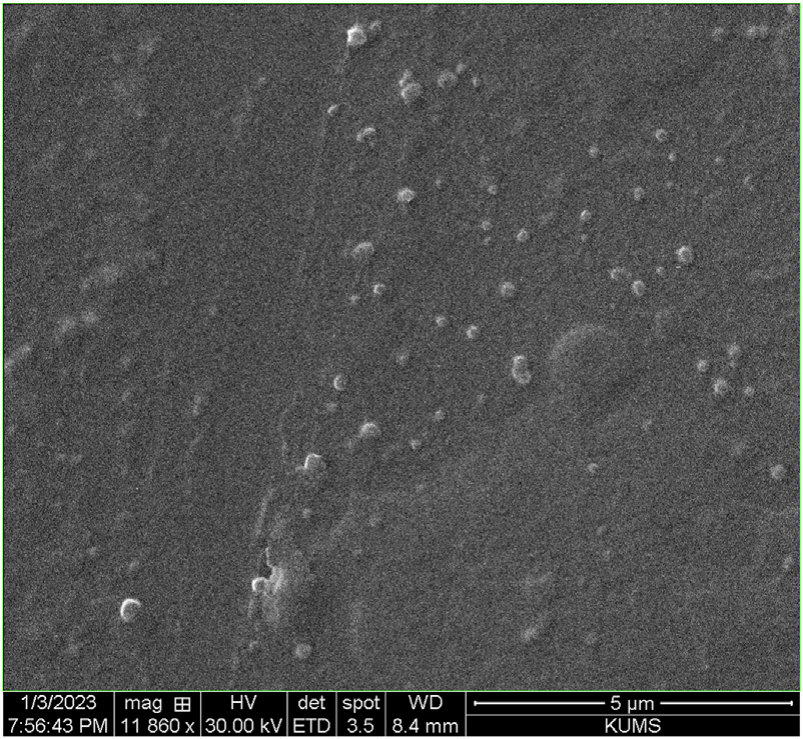
Scanning electron micrograph of insulin-loaded chitosan nanoparticles confirming the narrow size distribution and uniform spherical shape of the nanoparticles.

The highest possible Cs/insulin volume ratio without a deleterious effect on the size and PDI of the NPs and minimal effect on the EE was selected to synthesize the NPs. Accordingly, the ratio of 1:0.75 led to a hydrodynamic diameter of 165.3 nm and PDI of 0.24, which were in an excellent range for an IN formulation. According to these values, a narrow size range distribution results in stability, homogeneity, and less aggregation (Figure 1A). In addition, the zeta potential was +21.6 mV (Figure 1B). Moreover, SEM analysis of insulin-loaded Cs-NPs confirmed that the NPs were spherical and had a uniform size distribution (Figure 2).

**FIGURE 3:**
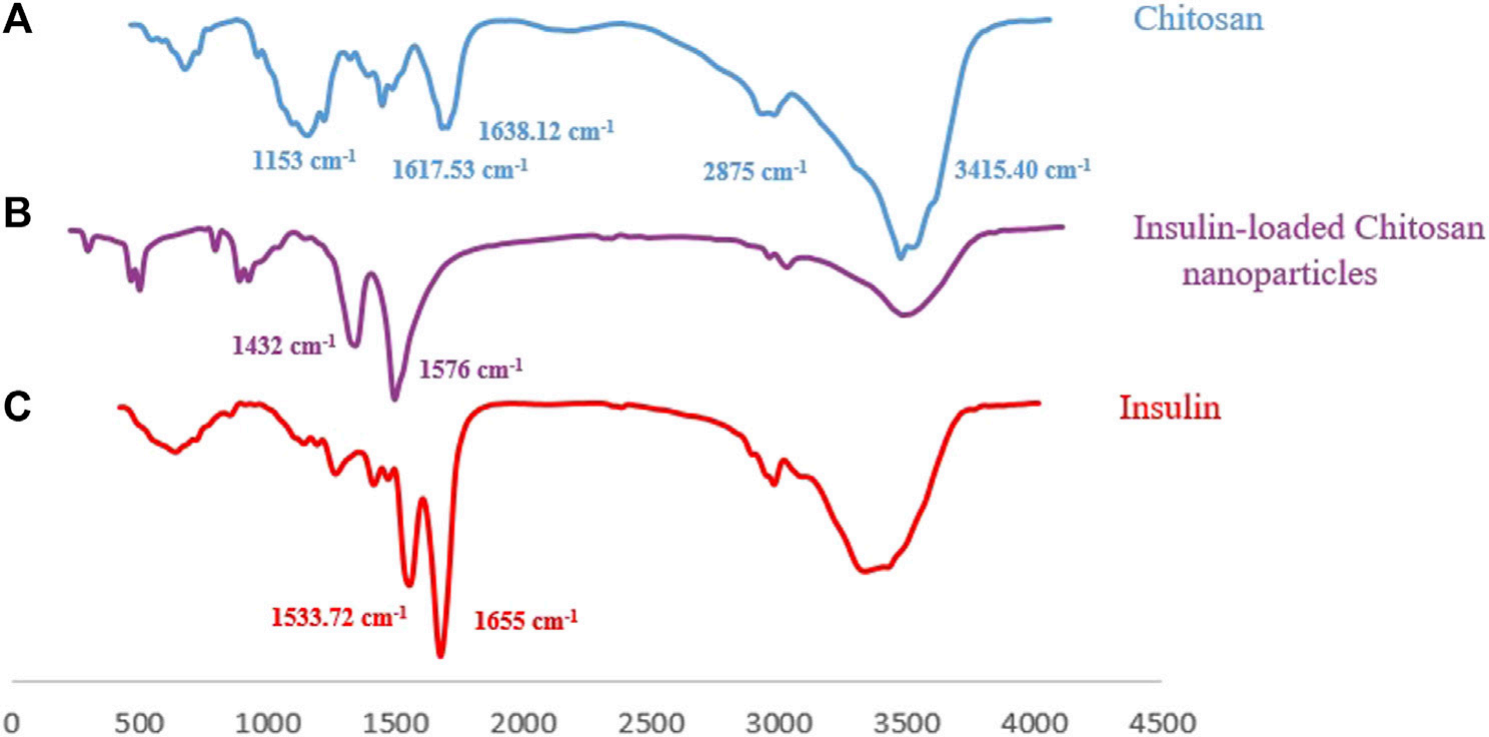
**(A)** Fourier-transform infrared spectrum of chitosan with its characteristic peaks, **(B)** FT-IR spectrum of the insulin-loaded chitosan nanoparticle implying an effective interaction between Cs and insulin, and **(C)** FT-IR spectrum of insulin with its characteristic peaks.

Furthermore, the FT-IR spectra of pure Cs, insulin-loaded Cs-NPs, and insulin are illustrated in Figures 3A–C, respectively. The functional peaks of Cs were at 3415.40 cm^−1^ (OH stretching), 2875 cm^−1^ (CH stretching), and 1638.12 cm^−1^ (amide I; Figure 3A). The spectrum of Cs exhibited an amine deformation peak at 1617.53 cm^−1^ and an amide I carbonyl stretch at 1638.12 cm^−1^ (Figure 3A). In the spectrum of the insulin-loaded Cs-NPs, the amine deformation peak of Cs shifted from 1617.53 cm^−1^ to 1432 cm^−1^, and the peak at 1638.12 cm^−1^ changed into a sharp peak at 1576 cm^−1^ meant an interaction was occurring at the amine groups on the Cs, and this can correspond to the binding of insulin to these sites (Figure 3B). The insulin spectrum expressed distinct shoulder absorptions in the amide I (1655 cm^−1^) and amide II (1533.72 cm^−1^) regions as characteristic of typical protein spectra (Figure 3C). In addition, the peak intensity of amino groups of Cs at 1153 cm^−1^ was decreased after complexation with insulin (Figure 3C). The characteristic band of insulin at 1655 cm^−1^ and the Cs at 1638 cm^−1^ did not appear in the insulin-loaded Cs-NP spectra because they were masked due to the physical interaction between Cs and insulin. Hence, the FT-IR results presented here suggested that a successful interaction has occurred between the polymer and drug after complexation driven by opposite-charged Cs and insulin.

**FIGURE 4:**
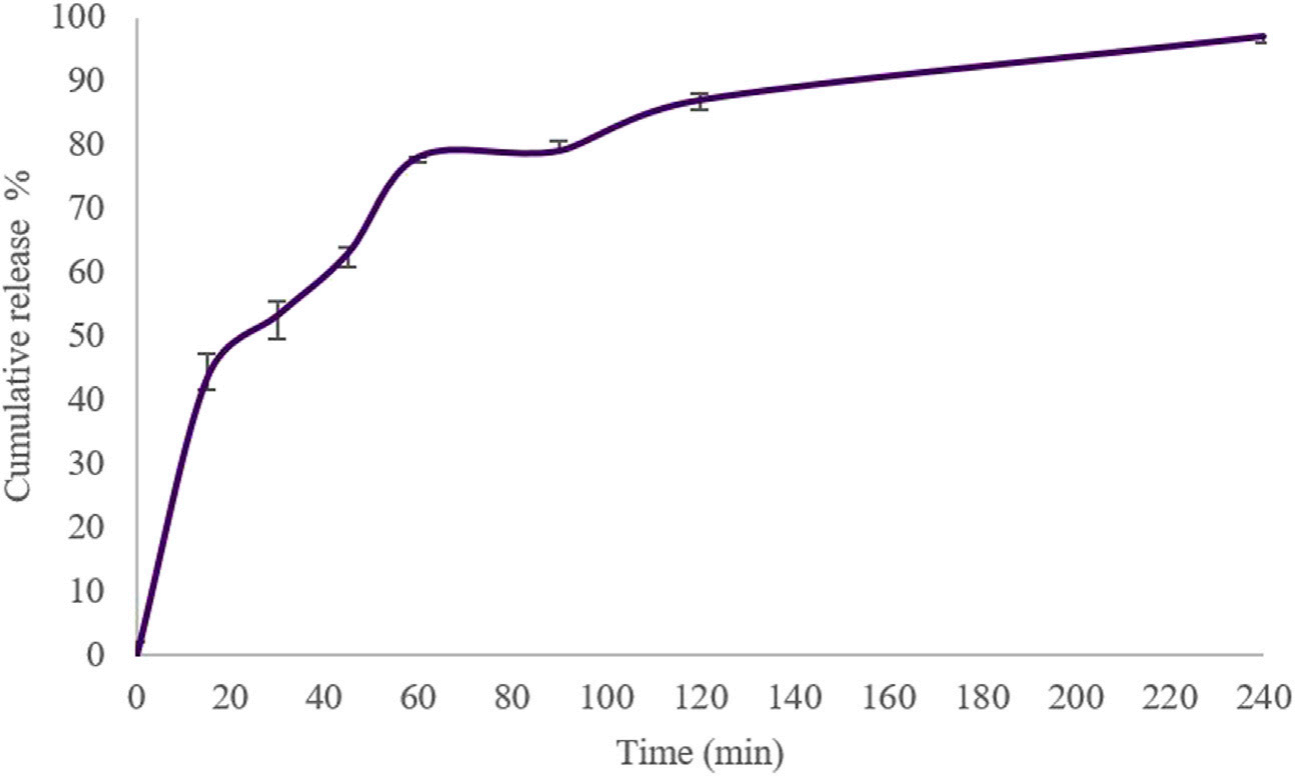
*In vitro* cumulative release of insulin from chitosan nanoparticles in the simulated nasal fluid (pH 6.5 ± 0.1), 0–240 min, at 33 °C ± 2 °C and constant shaking.

The cumulative amount of insulin released from Cs-NPs was plotted against time for 4 h. Our findings showed that the release of insulin from Cs-NPs was more than 90% within this time interval in a biphasic drug release pattern from Cs-NPs, with a burst release occurring within the first 15 min and a gradual release over 4 h (Figure 4). A mathematical model describing the drug release from the Cs-NPs was the Korsmeyer–Peppas model, as the cumulative release best fitted in this model (*R* ^2^ = 0.9792; Supplementary Figure S1), in comparison to zero order (*R* ^2^ = 0.8528; Supplementary Figure S2A), Higuchi (*R* ^2^ = 0.9533; Supplementary Figure S2B), first order (*R* ^2^ = 0.931; Supplementary Figure S2C), and Hixson (*R* ^2^ = 0.9065; Supplementary Figure S2D).

### 3.3 *Ex vivo* diffusion evaluation of insulin-loaded Cs-NPs in SNF and KHB solutions

**FIGURE 5:**
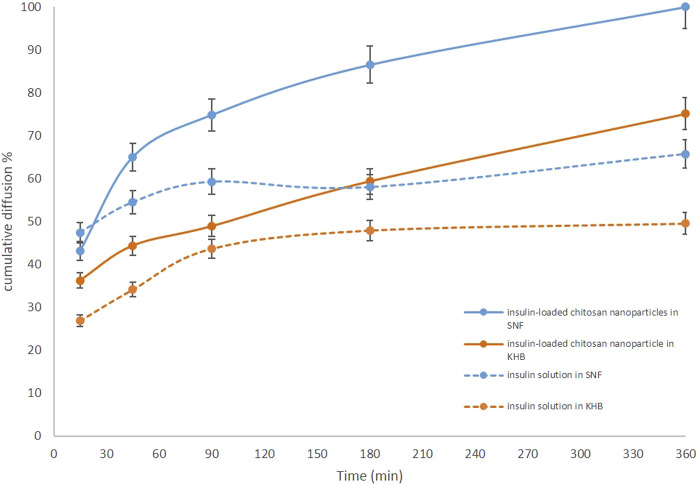
Comparison of the *ex vivo* diffusion rates of insulin solution and insulin-loaded chitosan nanoparticles from a freshly isolated ovine nasal tissue in the Krebs–Henseleit buffer (orange solid and dashed lines) and simulated nasal fluid (blue solid and dashed lines).

Here, the KHB (268 mOsmol) was compared to the SNF (391 mOsmol), as the glucose-free medium was commonly employed for these types of assays (Table 1). The feasibility of using the KHB instead of the SNF in the *ex vivo* models for IN formulation studies was evaluated for both insulin-loaded Cs-NPs and insulin solution. For the insulin solution, initial diffusion (20%) for the SNF- and KHB-filled apparatus was 0.06 and 5.80 min, respectively. However, the half diffusion rate (50%) was 21.89 min and 289.32 min for SNF- and. KHB-filled apparatus, respectively. Additionally, the inferring values for 70% of the diffusion rate of insulin in the SNF and KHB apparatus from the extrapolating of data were estimated to be 529.29 h and 3251.91 h, respectively (Supplementary Table S1). Similarly, the time of initial diffusion (20%) for insulin-loaded Cs-NPs in the SNF-filled apparatus was 3.83 min in comparison to the KHB-filled apparatus, which was 5.04 min. The diffusion rate of 50% occurred at 21.23 and 65.35 min for SNF and KHB groups, respectively. Furthermore, 70% of the insulin diffusion using insulin-loaded Cs-NPs was 66.45 and 360.30 min for SNF and KHB groups, respectively (Supplementary Table S2). This trend analysis for the SNF and KHB showed an approximately similar pattern in diffusion data over time for both the insulin solution and insulin-loaded Cs-NP diffusion which confirmed the validity of KHB usage instead of the SNF for *ex vivo* IN diffusion studies (Figure 5).

**TABLE 1:** Compositions of the simulated nasal fluid (pH 6.4) and Krebs–Henseleit buffer solution (pH 7.4).

Buffer solution	Composition mM (mOsmol)						Total (mOsmol)
	NaCl	KCl	CaCl_2_	MgSO_4_	KHPO_4_	D-glucose	
**Simulated nasal fluid**	150.061 (300.12)	39.97 (79.94)	3.94 (11.83)	-	-	-	391.89
**Krebs–Henseleit buffer solution**	118 (236)	4.8 (9.6)	2.5 (7.5)	1.2 (2.4)	1.2 (2.4)	11 (Vitore et al., 2023)	268.9

**FIGURE 6:**
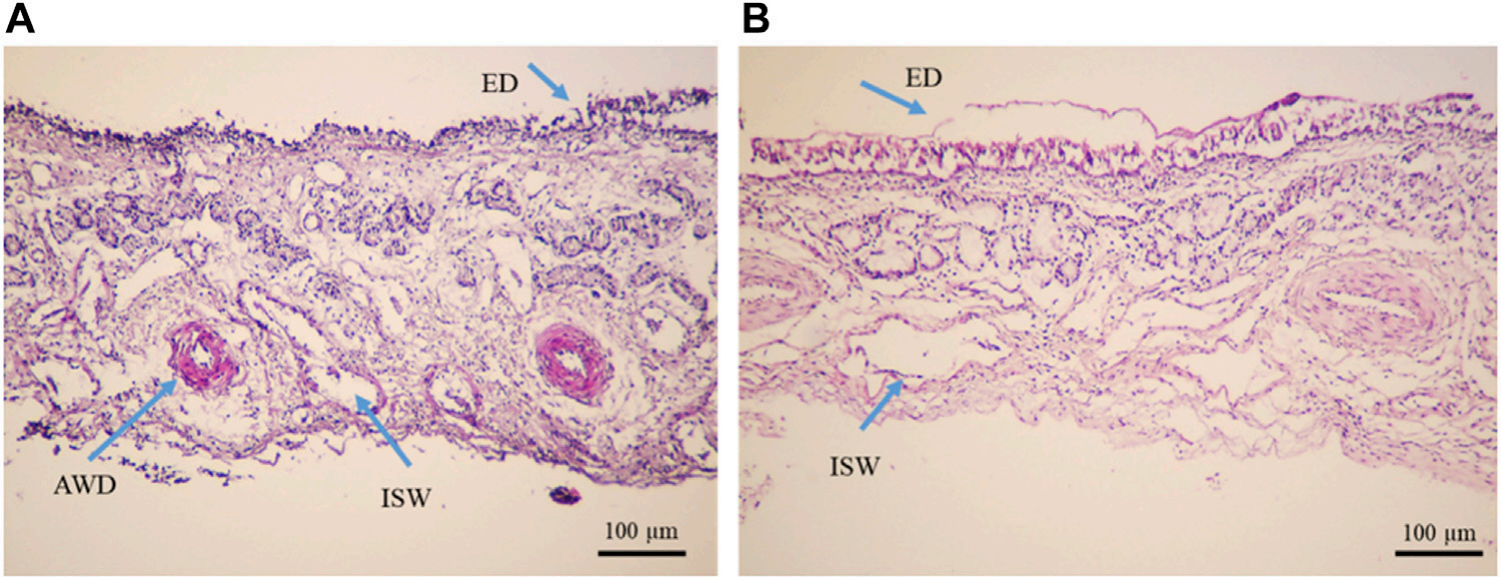
Histopathological analysis of tissue mounted on Franz’s cells filled with the **(A)** Krebs–Henseleit buffer solution (pH 7.4) and **(B)** simulated nasal fluid (pH 6.4), after a 4-h *ex vivo* diffusion test, under the light microscope (Olympus, Japan), ×10 magnification. The yellow arrows show epithelial detachment (ED), arterial wall damage (AWD), venule wall damage (VWD), and intercellular space widening (ISW).

Moreover, the results of both diffusion experiments proved the effectiveness of the Cs-NPs in comparison to the insulin solution in the IN route of administration. The diffusion efficacy of insulin in the insulin-loaded Cs-NP groups was compared to the insulin solution and showed increased insulin diffusion (solid lines vs. dashed lines) in both buffer solutions. Diffusion rates for both insulin-loaded Cs-NPs and insulin solution confirmed the validity of KHB utilization instead of the SNF for *ex vivo* intranasal diffusion studies. It is notable that a smaller amount of cumulative diffusion rate was recorded for the KHB group in all the measurement points. Furthermore, the deviation between SNF and KHB groups increased during the diffusion time, due to the higher diffusion in the SNF groups that reached the maximum value in the latest part of the experiment (Figures 6A,B). Moreover, we evaluated the histological indications of SNF- and KHB-affected membranes to validate this methodology as an alternative to the conventional *ex vivo* test using the SNF. In this perspective, there is no previous comparable study that relates to the cell viability of the OVINAE.

### 3.4 Statistical evaluation of SNF and KHB toxicity on the OVINAE

The inspection of the histopathological tissue samples mounted on Franz’s cell prefilled with the KHB and SNF revealed that lesions were distributed in both groups and variances were homogeneous as assessed for equality of variances (Supplementary Table S3). Therefore, we concluded that there are no statistically significant differences between the means. An independent *t*-test was run on the data with a 95% confidence interval (CI) for the means differences of the ED, AWD, VWD, and ISW to establish whether there were differences in these variables between KHB and SNF groups. The results revealed no difference between the SNF (2.3488 ± 0.16) and KHB (2.2000 ± 0.11) for the ED (*t* = 0.767; degree of freedom (df) = 106; *p* = 0.447); non-significant difference between the SNF (2.2558 ± 0.12) and KHB (2.00 ± 0.11) for the VWD variant (*t* = 1.460; df = 106; *p* = 0.147); a minor difference between the SNF (2.1860 ± 0.14977) and KHB (1.3846 ± 0.10212) for the AWD (*t* = 4.583; df = 106; *p* = .000); but a relatively meaningful difference between the SNF (3.00 ± 0.14119) and KHB (4.00) for the ISW (*t* = −8.729; df = 106; *p* = 0.00). The 95% CI of the difference between means did not indicate an overall alternation of tissue between the means of the SNF and KHB samples (Table 2). The histopathological observations of the nasal mucosa statistically confirmed that the KHB is a practical milieu supporting the viability of the OVINAE in comparison to the SNF.

**TABLE 2:** Lesion frequencies of ovine nasal epithelium tissues used in two supporting solution media.

Lesion	Simulated nasal fluid	Krebs–Henseleit buffer solution
**Epithelial detachment**	2.34 ± 0.162	2.20 ± 0.116
**Arterial wall damage**	2.18 ± 0.149	1.38 ± 0.102
**Venules wall damage**	2.25 ± 0.129	2.00 ± 0.113
**Intercellular space widening**	3.00 ± 0.129★	4.00 ± 0.000

★Significant difference at *p* ≤ 0.05.

### 3.5 Evaluation of the toxicity of the insulin-loaded Cs-NPs on the OVINAE

**FIGURE 7:**
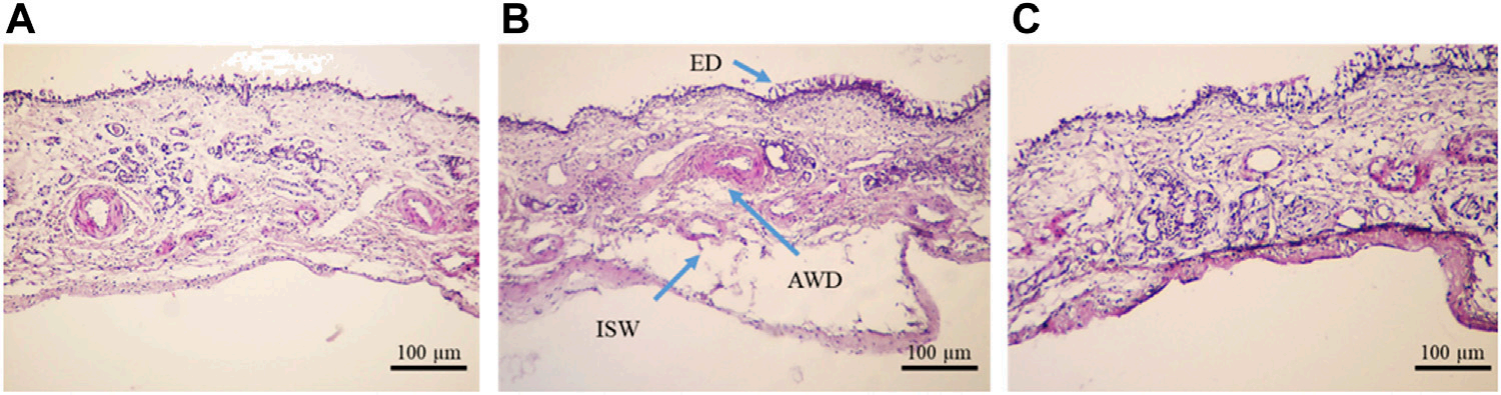
Ovine nasal epithelium tissue after 1 h incubation in the **(A)** negative control (NC), **(B)** positive control (PC), and **(C)** insulin-loaded chitosan nanoparticle stained with hematoxylin–eosin and observed under a light microscope (×10 magnification).

The histological sections of OVINAE samples stained with HE are shown in Figure 7. A sample of the NC, incubated in PBS, was not disintegrated structurally, and the whole structure of tissue remained intact without considerable morphological changes of the mucosa. However, some minor changes including mural destructive lesions of venules and disintegration of tissue were seen (Figure 7A). On the other hand, the nasal mucosa treated with isopropyl alcohol, PC, showed overtly delocalization of the epithelial layer from the beneath layer. Additionally, in some parts, arteriole walls were destroyed, small venules were widely damaged, and mural destructive lesions of venules occurred. In comparison to other groups, mural destructive lesions of venules are the most prominent aspect of the tissue in the PC treatment. However, ED was scarce in this group (Figure 7B). Instead, all the toxicity indicators including ED and AWD were not observed in the sample treated with insulin-loaded Cs-NPs, and the basal membrane as well as the superficial part of the submucosa remained unaffected. Nevertheless, mural mild destructive lesions of venules were seen in some sections in this group (Figure 7C). Overall, the structure of tissue was conserved in comparison to all other groups ensuring the efficacy, safety, and tolerability of insulin-loaded Cs-NPs for nasal mucosa.

Table 3 shows the toxicity statistics of the OVINAE samples after 1 h incubation in the NC, PC, and insulin-loaded Cs-NP samples. In all three groups, lesions were recorded after evaluation. In this context, for ED, the difference between all the groups was negligible. On the other hand, the assessment of the toxicity indications, including the ISW, AWD, and VWD, demonstrated that the NP group was similar to the NC group and significantly different from the PC group. This indicated the safety and tolerability of insulin-loaded Cs-NPs for the OVINAE.

**TABLE 3:** Toxicity indicators of the ovine nasal epithelium incubated in the insulin-loaded chitosan nanoparticle sample, negative control, and positive control.

Lesion	NC	PC	NPs
Epithelial detachment	2.5106 ± 0.11^ab^	2.9259 ± 0.19^b^	2.0476 ± .0.20^**a**^
Arterial wall damage	1.6383 ± 0.10^a^	2.8889 ± 0.15^b^	1.4762 ± 0.11^a^
Venule wall damage	1.2553 ± 0.06^a^	3.8148 ± .076^b^	1.1905 ± 0.08^a^
Intercellular space widening	2.5106 ± 0.12^a^	3.1852 ± 0.16^b^	2.0952 ± 0.20^a^

NC, negative control; PC, positive control; NPs, insulin-loaded chitosan nanoparticles.

## 4 Discussion

### 4.1 Development of insulin-loaded Cs-NPs

The Cs-NPs were formed based on the complexation of the positively charged amino groups of Cs at pH lower than its isoelectric point (pI) and negatively charged functional groups of insulin at pH above its pI. The preparation method of insulin-loaded Cs-NPs was a relatively straightforward methodology based on the self-assembly of the polymer and the drug, avoiding harsh chemical solvents and conditions (Sarmento et al., 2006; Sadeghi A. et al., 2008; Shamsa et al., 2018; Chen et al., 2019). The optimization experiment results showed that the higher the insulin solution volume up to the equal amount to the Cs solution, the higher EE would be. However, it resulted in much lower zeta potential and higher sizes which were unwarranted for nasal absorption and, thus, omitted from the further steps (data not shown). The final pH of the insulin-loaded Cs-NP solution (6.00 ± 0.1) was suitable for IN administration because the natural pH of the nasal cavity is 5.5–6.5 (Chin et al., 2021).

The mucoadhesive properties of positively charged NPs are largely determined by their interaction with negatively charged mucus, primarily through their zeta potential (Abruzzo et al., 2021; Gao et al., 2023; Spleis et al., 2023). This interaction was heavily influenced by the ratio of Cs to insulin in the formulation. To increase the zeta potential, we used the highest possible Cs/insulin volume ratio without a deleterious effect on the size and PDI of the NPs and minimal effect on the EE. According to these values, a narrow size range distribution resulted in stability, homogeneity, and less aggregation (Figure 1A). Additionally, the high zeta potential (+21.6 mV) warranted the mucoadhesion of Cs-NPs to the nasal tissue.

The predicted drug diffusion (delivery) time from the nasal cavity to the whole brain tissue ranged from 30 min to 2 h, depending on the animal models, formulations, and tracer molecules (Crowe and Hsu, 2022). The drug reaches the deeper parts of the brain such as the hypothalamus at its maximum level in 30 min (Crowe and Hsu, 2022). Furthermore, the CNS clearance is accomplished in about 4 h (Crowe and Hsu, 2022). Therefore, these values give a cue to estimate the elapsed time for studying IN-administered formulations. Accordingly, the cumulative amount of insulin released from Cs-NPs was plotted against time for 4 h. Our findings showed that the release of insulin from Cs-NPs was more than 90% within this time interval.

Concerning the Cs-based DDSs, numerous studies have revealed the successful encapsulation of insulin using PEC and ion gelation methods (Bhattacharyya et al., 2018; Ibie et al., 2019; Liu et al., 2019; Rong et al., 2019; Rathore et al., 2020; Cui et al., 2022; Arpaç et al., 2023). In comparison with the recent studies, our findings are consistent with the previous investigation for insulin-loaded Cs-Dz13Scr nanoparticles with a size of 159.3 nm and PDI of 0.331 (Weng et al., 2020). This study resulted in a higher EE (88%), but lower zeta potential (−1.08 mV) (Weng et al., 2020). Additionally, Wong et al. reported insulin-loaded Cs-NPs with a scaled-up step, resulting in a size of 479 nm, PDI of 0.34, zeta potential of 14.47 mV, and EE of 88.71% (Wong et al., 2020). Nevertheless, since the common goal of these studies was to deliver insulin orally, the EE was a more important parameter than the size and zeta potential. On the other hand, Nojoki et al. used tween 80 as a surfactant in a film hydration method to synthesize Cs-transfersulin as an IN-DD. The particle size was reported as 137.9 ± 28.2 nm, PDI 0.20, zeta potential +23.4 mV, and EE and DL 65.1% ± 0.9% and 9.1% ± 0.4% (Nojoki et al., 2022), respectively. However, they did not manage an *ex vivo* experiment to study the diffusion behavior of the insulin-loaded Cs-NPs in comparison to the insulin solution (*vide infra*).

### 4.2 *Ex vivo* analysis of diffusion and toxicity

A novel *ex vivo* model of a freshly isolated OVINAE was utilized for permeation assay. Herein, the KHB in the receptor compartment was used since cell viability is guaranteed in the KHB solution to practically mimic the near-to-real condition. The glucose component of the KHB can preserve cell viability and, thus, tissue integrity during the procedure (Bailey and Ong, 1978). As shown in Figure 5, a slight variance in insulin diffusion rate may be presumably as a result of dissimilar osmolarity between the SNF and KHB and the changed tissue behavior toward these media. Moreover, the diffusion efficacy comparison of insulin in the insulin-loaded Cs-NP group and insulin solution showed increased insulin diffusion in both buffer solutions with a smaller amount of cumulative diffusion rate for the KHB group in all the measurement points. This deviation might be due to the different compositions and pH of the SNF and KHB, which can alter the tissue diffusion capacity due to ED resulting from the damaging condition for the tissue in the SNF group (Figure 6A). Conversely, the integrity and viability of the OVINAE in the KHB solution can be preserved due to the glucose supply and the mild pH which might increasingly hamper the insulin penetration in both insulin solution and insulin-loaded Cs-NP groups (Figure 6B). As shown in Table 1, the KHB contains MgSO_4_ and KHPO_4_ components. In this line, an array of studies showed the anti-corrosive and cytoprotective activities of MgSO_4_ (Koning et al., 2019; Mohammadi et al., 2020; Zhu et al., 2020). Moreover, MgSO_4_ helps the glucose consumption of the OVINAE during the *ex vivo* experiment of drug diffusion and inhibits cell starvation. The SNF cannot support the metabolism of the tissue during the experiment and would be corrosive due to the lack of the mucin and MgSO_4_.

The histopathological observations of the nasal mucosa, statistically confirmed that the KHB is a practical milieu supporting the viability of the OVINAE through glucose supply in comparison to the SNF which is a glucose-free medium. However, it is important to take into account that the KHB does not mimic the composition and the pH of nasal fluid, so the results may not fully translate the *in vivo* situation either. Another important aim is to modify SNF and KHB formulations that can imitate the critical features of the relevant biological fluid, while conserving the tissues, and keep the membranes viable during the procedures of DDS permeation. This work provides the foundation for further work on modified formulations of buffer solutions to conserve membrane integrity while investigating the DDS permeability.

The synthesized NPs were characterized considering the IN-DDS requirements. Results showed that insulin was encapsulated in Cs-NPs in the appropriate zeta potential, size, and PDI, suitable to submit to IN preclinical settings. The histological studies indicate that Cs-NPs can increase the delivery of insulin without any notable toxicity to the nasal tissue. Thus, insulin-loaded Cs-NPs can be utilized as potential IN-DDSs by longer residence time and sustained diffusion in the IN insulin delivery. In agreement with the previous common method, our novel *ex vivo* test showed that the designed IN-DDS could improve insulin permeability through the OVINAE.

## Data Availability

The original contributions presented in the study are included in the article/Supplementary Material, further inquiries can be directed to the corresponding authors.

## Abbreviations

DDSs, drug delivery systems; IN, intranasal; KHB, Krebs–Henseleit buffer; OVINAE, ovine nasal epithelium; ED, epithelial detachment; and ISW, intercellular space widening.
