# Supplementary material for: Synthesis of an insulin-loaded mucoadhesive nanoparticle designed for intranasal administration: focus on new diffusion media
Source: Front Pharmacol. 2023 Aug 28;14:1227423. doi: 10.3389/fphar.2023.1227423 (PMC10494546; doi:10.3389/fphar.2023.1227423)
Supplement: Supplementary file 1 [file DataSheet1.docx]

Supplementary Material

Synthesis of insulin-loaded mucoadhesive nanoparticle designed for intranasal administration: Focus on new diffusion media

**Tahereh Jamshidnejad-Tosaramandani^1,2,3^, Soheila Kashanian^1,4*,^ Isaac Karimi^2^,**

**Helgi B. Schiöth^4*^**

*** Correspondences:**Soheila Kashanian

Helgi B. Schiöth

[kashanian_s@yahoo.com](mailto:kashanian_s@yahoo.com)

[helgi.schioth@neuro.uu.se](mailto:helgi.schioth@neuro.uu.se)

# Supplementary Figures and Tables

**Figure S1.** The Korsmeyer-peppas model describes the drug release from the chitosan nanoparticles mathematically as the cumulative release best fitted in this model (R^2^= 0.9792)

**(d)**

**(c)**

**(b)**

**(a)**

**(a)**

**(a)**

**Figure S2.** The **A)** zero order model (R^2^= 0.8528), **B)** first-order model (R² = 0.931), **C)** Higuchi model (R^2^= 0.9533), and **D)** Hixson model (R^2^=0.9065), for the drug release from the chitosan nanoparticles, which are not describing the drug release from the chitosan nanoparticles as the cumulative release not fitted in these model properly.

**Table S1.** The comparison of the insulin cumulative diffusion time through the ovine nasal epithelium mounted on the Franz's cell pre-filled with the simulated nasal fluid (pH 6.4) and Krebs-Henseleit buffer solution (pH 7.4)

| Cumulative diffusion (%) | Time of diffusion in simulated nasal fluid (min) | Time of diffusion in Krebs-Henseleit buffer solution (min) |
| --- | --- | --- |
| 20 | 0.069732464 | 5.808077431 |
| 45 | 8.398208404 | 150.8301316 |
| 50 | 21.89459798 | 289.3205302 |
| 60 | 148.8118893 | 1064.538903 |
| 70 | 1011.435717 | 3916.912069 |
| 100 | 317570.5721 | 195115.0084 |

**Table S2.** The comparison of the insulin-loaded chitosan nanoparticle's cumulative diffusion time through the ovine nasal epithelium mounted on the Franz's cell pre-filled with the simulated nasal fluid (pH 6.4) and Krebs-Henseleit buffer solution (pH 7.4)

| Cumulative diffusion (%) | Time of diffusion in simulated nasal fluid (min) | Time of diffusion in Krebs-Henseleit buffer solution (min) |
| --- | --- | --- |
| 20 | 3.834277646 | 5.049416991 |
| 45 | 15.9629502 | 42.65339458 |
| 50 | 21.23239959 | 65.35759653 |
| 60 | 37.56392107 | 153.4549911 |
| 70 | 66.45731023 | 360.3014116 |
| 100 | 368.0088654 | 4663.594693 |

**Table S3.** The Levene's test results for equality of variances of the Krebs-Henseleit buffer solution and simulated nasal fluid influence on the epithelial detachment (ED), arterial wall damage (AWD), venules wall damage (VWD), and intercellular space widening (ISW) variables. The *F* value for all the variables is ≥ 0.05, except for the VWD.

| **Lesions** | | Levene's Test for Equality of Variances | | t-test for Equality of Means | |
| --- | --- | --- | --- | --- | --- |
|  |  | F | Sig. | t | df |
|  |  |  |  |  |  |
| ED | Equal variances assumed | 2.151 | 0.145 | 0.764 | 106 |
|  | Equal variances not assumed |  |  | 0.744 | 81.961 |
| AWD | Equal variances assumed | 2.226 | 0.139 | 4.583 | 106 |
|  | Equal variances not assumed |  |  | 4.421 | 78.935 |
| VWD | Equal variances assumed | 0.018 | 0.893 | 1.460 | 106 |
|  | Equal variances not assumed |  |  | 1.485 | 94.976 |
| ISW | Equal variances assumed | 88.606 | 0.000 | -8.729 | 106 |
|  | Equal variances not assumed |  |  | -7.083 | 42.000 |
